# Opposite Roles of NMDA Receptors in Relapsing and Primary Progressive Multiple Sclerosis

**DOI:** 10.1371/journal.pone.0067357

**Published:** 2013-06-28

**Authors:** Silvia Rossi, Valeria Studer, Alessandro Moscatelli, Caterina Motta, Giancarlo Coghe, Giuseppe Fenu, Stacy Caillier, Fabio Buttari, Francesco Mori, Francesca Barbieri, Maura Castelli, Valentina De Chiara, Fabrizia Monteleone, Raffaele Mancino, Giorgio Bernardi, Sergio E. Baranzini, Maria G. Marrosu, Jorge R. Oksenberg, Diego Centonze

**Affiliations:** 1 Clinica Neurologica, Dipartimento di Medicina dei Sistemi, Università Tor Vergata, Rome, Italy; 2 Fondazione Santa Lucia/Centro Europeo per la Ricerca sul Cervello, Rome, Italy; 3 Centro Sclerosi Multipla, Dipartimento di Sanità Pubblica, Medicina Clinica e Molecolare, Università di Cagliari, Cagliari, Italy; 4 Department of Neurology, University of California San Francisco, San Francisco, California, United States of America; 5 Clinica Oculistica, Dipartimento di Biopatologia, Università Tor Vergata,Rome, Italy; 6 Department of Cognitive Neuroscience and Cognitive Interaction Technology Center of Excellence, Bielefeld University, Bielefeld, Germany; Innsbruck Medical University, Austria

## Abstract

Synaptic transmission and plasticity mediated by NMDA receptors (NMDARs) could modulate the severity of multiple sclerosis (MS). Here the role of NMDARs in MS was first explored in 691 subjects carrying specific allelic variants of the NR1 subunit gene or of the NR2B subunit gene of this glutamate receptor. The analysis was replicated for significant SNPs in an independent sample of 1548 MS subjects. The C allele of rs4880213 was found to be associated with reduced NMDAR-mediated cortical excitability, and with increased probability of having more disability than the CT/TT MS subjects. MS severity was higher in the CC group among relapsing-remitting MS (RR-MS) patients, while primary progressive MS (PP-MS) subjects homozygous for the T allele had more pronounced clinical worsening. Mean time to first relapse, but not to an active MRI scan, was lower in the CC group of RR-MS patients, and the number of subjects with two or more clinical relapses in the first two years of the disease was higher in CC compared to CT/TT group. Furthermore, the percentage of relapses associated with residual disability was lower in subjects carrying the T allele. Lesion load at the MRI was conversely unaffected by the C or T allele of this SNP in RR-MS patients. Axonal and neuronal degeneration at the optical coherence tomography was more severe in the TT group of PP-MS patients, while reduced retinal nerve fiber thickness had less consequences on visual acuity in RR-MS patients bearing the T allele. Finally, the T allele was associated with preserved cognitive abilities at the Rao’s brief repeatable neuropsychological battery in RR-MS. Signaling through glutamate NMDARs enhances both compensatory synaptic plasticity and excitotoxic neurodegeneration, impacting in opposite ways on RR-MS and PP-MS pathophysiological mechanisms.

## Introduction

Glutamate is the main excitatory neurotransmitter in the mammalian brain, responsible for basal excitatory synaptic transmission and for many forms of synaptic plasticity. In certain neurological diseases, the stimulation of glutamate NMDA receptors (NMDARs) has the potential of limiting the clinical manifestations of neuronal damage by promoting compensatory plasticity in surviving neurons [1]–[5], but sustained activation of these receptors can be per se neurotoxic [6]–[11].

In multiple sclerosis (MS), NMDAR-dependent adaptive plasticity and excitotoxic neurodegeneration could both play a role in disease expression. In fact, in relapsing-remitting MS (RR-MS), a certain degree of inflammatory white matter damage is generally well tolerated, and reversible or irreversible clinical disability only appears when the adaptive abilities of the brain fail. Instead, in primary progressive MS (PP-MS) accumulating disability reflects the progression of neuronal damage without any compensation by plasticity mechanisms exhausted during clinically silent inflammatory episodes [12]–[14]. Whether NMDARs are directly involved in MS pathophysiology is however unknown.

Functional NMDARs are composed of a NR1 subunit (GRIN1), and one of the four NR2 subunits (GRIN2A, GRIN2B, GRIN2C and GRIN2D) [2], [15], [16]. The 5different subunits determine the binding properties of the receptor, and its permeability to extracellular cations, as well as the interaction of the receptor with intracellular scaffolding, anchoring and signaling molecules [8]. Specific single nucleotide polymorphisms (SNPs) of GRIN1 and GRIN2B subunits have been recently suggested to alter NMDAR function [17], and their possible association with MS could therefore inform on the role of NMDARs in both plasticity and neurodegeneration mechanisms occurring in this disease.

Thus, in the present study the role of NMDARs in the MS phenotype was explored in subjects carrying specific allelic variants in the NR1 subunit gene (GRIN1 rs4880213 and rs6293) or the NR2B subunit gene (GRIN2B rs7301328 and rs1805247). Consistent with a dual role of NMDARs in compensatory plasticity and neurodegeneration, we found that the T allele of rs4880213 SNP is associated with increased brain excitability in MS patients and with milder and severer RR-MS and PP-MS, respectively.

## Results

### Clinical Characteristics of MS Patients

Demographical features and clinical characteristics of the two samples of MS patients are shown in table 1. The MS sample used in replication analisys was different in terms of disability, disease duration, proportion of males and PP subjects.

**Table 1:** Demographic and clinical characteristics of MS subjects.

	discovery dataset	replication dataset	p value
Number	691	1548	
sex (M/F)	229/462	407/1141	<0.01
age at onset (yrs)	31.12±9.77	28.22±9.28	0.15
disease duration (yrs)	11.91±7.93	9.83±8.78	<0.01
onset (R/P)	613/78	1449/99	<0.01
EDSS	2.42±1.90	3.54±2.11	<0.01

M, male; F, female; yrs, years; R, relapsing; P, progressive; MSSS, Multiple.

Sclerosis Severity Scale; EDSS, Expanded Disability Status Scale.

The last Expanded Disability Status Scale (EDSS) available was <2.0 in 45.3% of patients of discovery dataset and in 19.8% of patients of replication dataset. The minimum and maximum EDSS values were respectively 0 and 8.0 for both samples.

All RR-MS patients had received immunomodulatory treatment during the course of their disease. First-line treatment was started for all patients at the time of the diagnosis. A percentage of patients (46%) had two immunomodulatory treatments. Patients with PP-MS did not receive immunomodulatory or immunosuppressive treatments.

### Prognostic Models

We assessed the impact of the four polymorphisms located in genes coding for NMDAR subunits on neurological disability of our group of MS subjects (discovery dataset) with two different models.

First, we evaluated the association of each single SNP with the EDSS by means of the Kruskal-Wallis rank sum test. The EDSS was significantly associated with the rs4880213 polymorphism (corrected p-value  = 0.02), whereas there were no significant association with the other three polymorphisms (not shown). Then, we applied a multiple logistic regression, using the dichotomous EDSS as response variable (cut-off point of 2.0) and the variables described in the method session as predictors. For each polymorphism, we used the number of copies of the minor allele as the predictor variable. The minor allele is T in rs4880213, C in rs7301328, G in rs1805247, and G in rs6293. The estimates of the parameters, Standard errors (SE), and related p-values (Wald statistics) are summarized in Table 2. The model returned a statistically non-zero coefficients for the SNP rs4880213, in addition to the clinical variables onset type, age and duration of the disease, and of the site of recruitment - a statistically non-zero coefficient means that the response variable (dichotomous EDSS in our model) is significantly affected by the predictor variable. The likelihood ratio test confirmed the significant association between the SNP rs4880213 and the severity of the disease; the model including the polymorphism fits the data significantly better than the model non-including the polymorphism (Chi squared = 0.7, DF = 1, p  = 0.001). Overall, the logistic regression predicts that, at equal values of age, disease duration, onset type, gender and the other SNPs, the probability of having an EDSS greater or equal 2.0 decreases as the number of copies of T in rs4880213 increases (0 ->1 ->2).

**Table 2:** Logistic regression (dichotomous EDSS as response variable, 0 if EDSS <2.0).

	Estimate	SE	z value	uncorrected p	corrected p
years of disease	0.07	0.02	4.13	<0.001	<0.001
gender (M)	0.43	0.20	2.17	0.03	0.12
onset (R)	−4.93	1.41	−3.49	<0.001	<0.001
age at sample	0.05	0.01	4.49	<0.001	<0.001
site (Rome)	1.06	0.25	4.29	<0.001	<0.001
rs4880213 (T)	−0.47	0.15	−3.20	<0.001	0.01
rs6293 (G)	−0.19	0.15	−1.28	0.20	0.60
rs1805247 (G)	0.12	0.25	0.48	0.63	1.00
rs7301328 (C)	0.03	0.14	0.20	0.84	1.00
rs4880213 (T) (replication dataset)	−0.26	0.10	−2.64	0.01	0.04

In the last two columns, the uncorrected p-values and the corrected p-values (Holm correction). The p-value for the replication data set is adjusted together with the other variables of the discovery data set.

SE, standard error; R, relapsing.

The association between rs4880213 SNP and disease severity was replicated in an independent sample (replication dataset), as shown in table 2. Also in the replication dataset, the multiple logistic regression confirmed the significant association between the rs4880213 polymorphism and the severity of the disease. The probability of having an EDSS greater or equal 2.0 decreases as the number of copies of T in rs4880213 increases. In this first logistic model, we chose the EDSS value of 2.0 as cut-off point of early clinical disability because of sample distribution, only a minor part of the discovery data set having higher EDSS scores. In order to confirm the result for more clinically significant values of EDSS, we pooled the two data sets and replicated the analysis using EDSS >4.0 as cut-off value. This alternative model confirmed the association between the SNP rs4880213 and the severity of the disease (Estimate = −0.18, SE = 0.07, z value = −2.39, Wald uncorrect p = 0.02, Holm corrected p = 0.03).

### rs4880213 T allele Increases Cortical Excitability in MS Patients

As we have already done in healthy individuals [17], we explored in a subgroup of MS patients (n = 84, 56 female, age 39±9 years, EDSS: 2.6±1.2) the impact of allelic variants of the four NMDAR SNPs in cortical excitability explored through paired TMS experiments.

**Figure 1:**
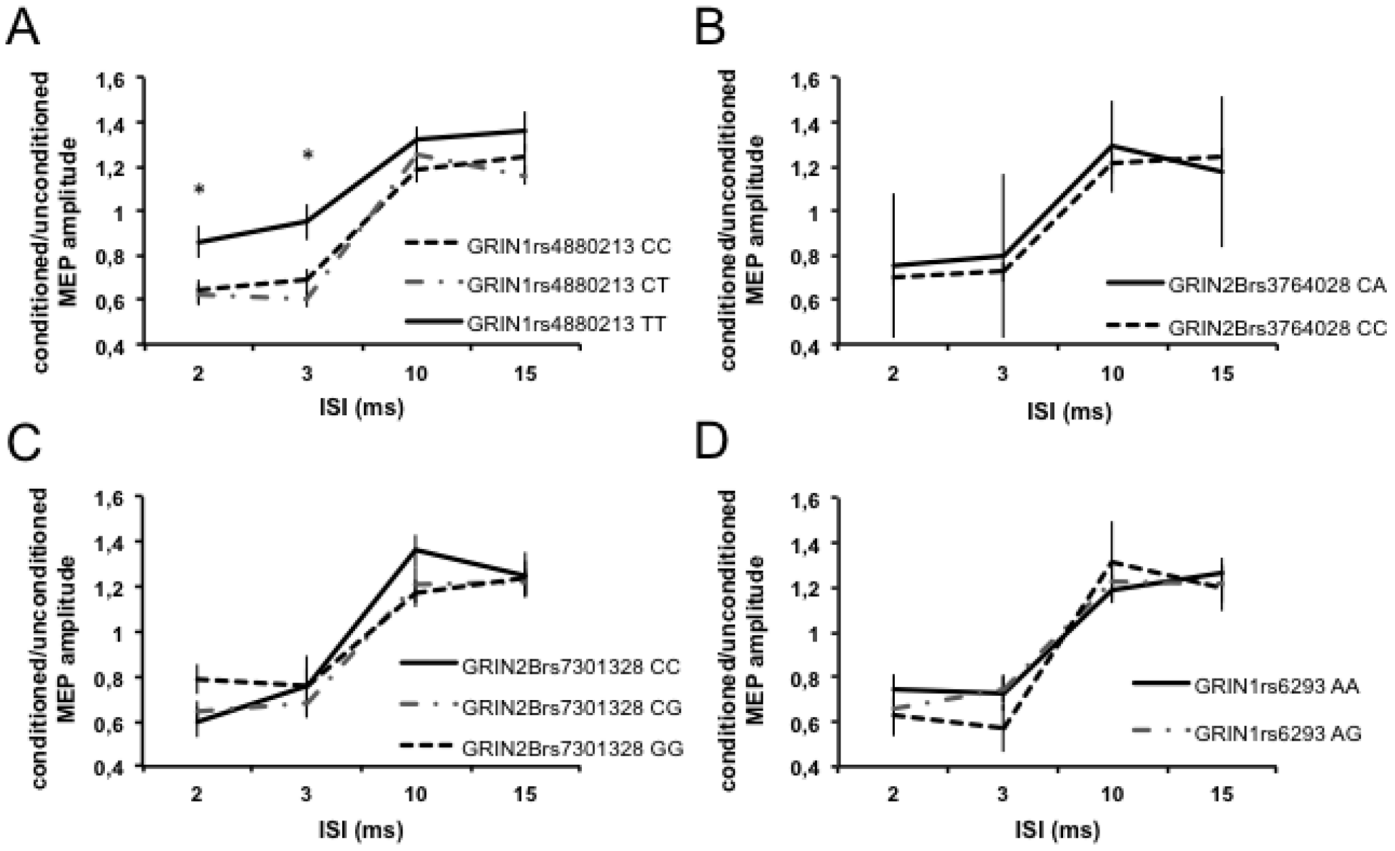
Effect of SNPs in the GRIN1 and GRIN2B genes on cortical excitability in MS. Role of A) GRIN1 rs4880213 polymorphism in the regulation of cortical excitability. In subjects carrying the ‘TT’ genotype, SICI was significantly lower than in the other subjects at 2 and 3 ms interstimulus intervals (ISI). No significant effect of B) GRIN2B rs1805247, C) GRIN2B rs7301328 and D) GRIN1rs6293 SNPs was found on SICI/ICF. In the four panels, the x-axis indicates the ISI in ms, the y-axis shows the MEP size elicited by paired-pulse stimulation. * means p<0.05.

The TMS procedure was well tolerated by all subjects. Mean resting motor threshold (RMT), active motor threshold (AMT), and motor evoked potential (MEP) latencies were not significantly different among “CC”, “CT”, and “TT” subjects of the rs4880213 SNP (Table 3), suggesting that these variants do not affect the intrinsic excitability of cortical neurons [19]. We then addressed the possible role of SNP rs4880213 in the regulation of synaptic excitability by means of paired TMS experiments. Analysis was performed using a repeated measures ANOVA on the normalized data with genotype as between-subjects and ISI as within-subject main factors. The analysis showed a significant main effect of ISI (F = 29.35, p<0.05) and genotype (F = 3.45, p<0.05) and a significant genotype x ISI interaction (F = 3.56, p<0.05) on short-interval intracortical inhibition (SICI) values. Post hoc contrasts revealed that the “TT” group had less SICI than the other two genotypic groups at ISIs 2 ms and 3 ms. Intracortical facilitation (ICF) showed a significant effect of ISI (F = 18.04, p<0.01), but no significant effect of genotype and genotype x ISI interaction. In contrast, subjects homozygous and heterozygous for the other GRIN1 or GRIN2B SNPs did not differ for their response to TMS at any ISI evaluated (Fig. 1).

**Table 3:** Mean RMT, AMT, and MEP latency were not significantly different among CC, CT, and TT subjects of the rs4880213 SNP.

GRIN1rs4880213	number	MEP latency	RMT	AMT
CC	24	22.55±0.62	47.17±1.60	35.91±1.44
CT	42	22.38±0.41	46.80±1.42	35.09±1.35
TT	18	23.57±0.79	48.45±2.43	36.55±2.55

MEP, motor evoked potential; RMT, resting motor threshold; AMT, active motor threshold.

### Opposite Effects of rs4880213 T allele on Disability Progression in RR-MS and PP-MS

**Figure 2:**
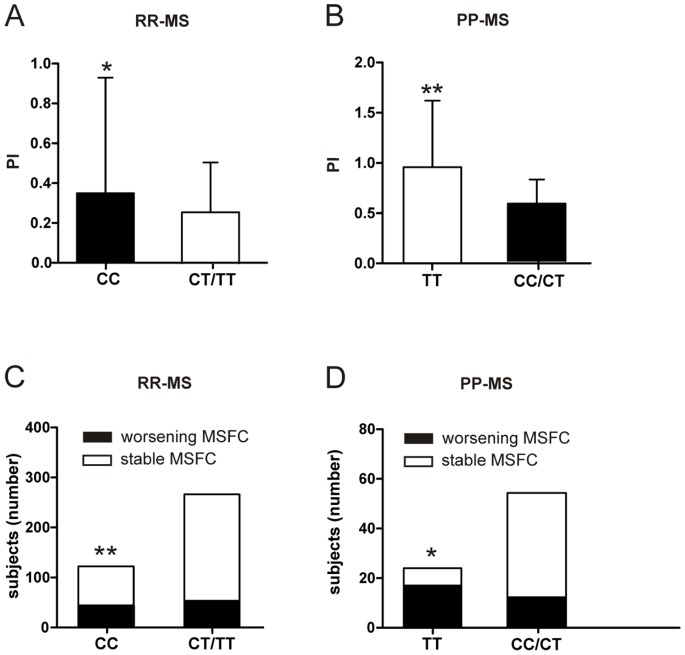
Effect of SNP rs4880213 on disability progression in MS. A. The graph shows that PI was lower among RR-MS patients with T allele. B. PI was higher among PP-MS subjects homozygous for T allele. C. Disability progression at MSFC was less frequent among RR-MS patients with T allele. D. The graph shows that early disability progression at MSFC (in the first two years of the disease) was more frequent among PP-MS subjects homozygous for T allele. * means p<0.05; ** means p<0.01.

The increased excitatory glutamatergic transmission demonstrated in MS subjects with rs4880213 T allele, could permit synaptic plasticity and recovery from disability, thus explaining the protective effects on EDSS worsening. Further analyses were conducted to test this hypothesis, stratifying by genotype and onset (Table 4). Remarkably, an opposite statistically significant difference was found by analyzing direct measures of disability. Mean progression index (PI) (p = 0.03; Fig. 2A) and mean MS severity score (MSSS) (2.98±2.5 versus 2.39±2.2, p<0.01) were higher in CC group among RR-MS subjects, in line with the primary overall analysis. Conversely, mean PI (p<0.01; Fig. 2B) and mean MSSS (7.96±1.1 versus 6.54±1.2, p<0.001) were higher in TT group among PP-MS subjects, suggesting that increased NMDAR-mediated excitatory transmission could favor disability progression in PP-MS. A logistic regression analysis confirmed the significant association between TT genotype and the probability of having an EDSS greater than 4.0 for PP-MS subjects, at equal values of age, disease duration, and gender (OR = 13.5, 2.3–78.1; coefficient = 2.6, SE = 0.9, p = 0.003).

**Table 4:** Characteristics of MS subjects (discovery dataset) according to SNP rs4880213.

	RR-MS			PP-MS		
genotype	CC	CT	TT	CC	CT	TT
number	130	210	74	22	32	24
sex (M/F)	34/96	61/149	20/54	12/10	10/22	10/14
age at onset (yrs)	29.53±8.5	29.87±8.9	31.65±10.9	39.90±13.5	41.37±10.5	39.66±7.3
disease duration (yrs)	13.10±7.5	11.48±8.4	11.52±9.2	10.90±4.7	9.15±4.5	8.75±6.0

RR-MS, relapsing remitting multiple sclerosis; PP-MS, primary progressive multiple sclerosis; M, male; F, female; yrs, years.

Similar results were obtained when analyzing MS functional composite (MSFC) scores. Disability progression at MSFC was less frequent among RR-MS patients with T allele (19.3% versus 35.0%, p = 0.001) (Fig. 2C). Of note, timed walk test (TWT) and nine hole peg test (9HPT) baseline values were not significantly different between the two groups (TWT: CC 8.90±3.9 s, CT/TT 8.71±7.7 s, p = 0.80; 9HPT: CC 23.60±7.1 s, CT/TT 22.82±5.9 s, p = 0.26), but paced auditory serial addition test (PASAT) baseline mean score was higher in CT/TT group (43.61±13.0 versus 36.34±11.3, p<0.001), again suggesting the beneficial effects of an increase excitatory synaptic transmission on neuronal function in MS-RR. Conversely, the proportion of PP-MS patients with an early MSFC worsening was significantly higher in TT group (2 year disease duration: 70.8% versus 22.2%, p = 0.03; 5 year disease duration: 91.6% versus 72.2%, p = 0.07) (Fig. 2D). Early change in TWT has been recently showed as a strong predictor of long-term disability in progressive MS [20]. The frequency of PP-MS subjects with TWT worsening was significantly higher in TT group (2 year disease duration: 58.3% versus 16.6% p<0.01; 5 year disease duration: 91.6% versus 53.7%; p<0.01).

Finally, Bayesian Risk Estimate for MS (BREMS) scores were calculated to identify individual risk of secondary progression [21] for RR-MS patients. The percentage of patients with BREMS score ≤-0.63 was significantly higher in CT/TT group (10.6%) than in the CC group (2.5%, p = 0.007), again confirming the protective effects of rs4880213 T allele in RR-MS. The frequency of patients with BREMS score ≥2 was not significantly different between the two groups, although lower among subjects with T allele (6.0% versus 10.8%, p = 0.14). Patients with BREMS score ≥2 are considered at very high risk of secondary progression while patients with BREMS score ≤-0.63 are very likely to remain progression free [21].

### rs4880213 T allele Reduces the Clinical and MRI Impact of Disease Activity in RR-MS

**Figure 3:**
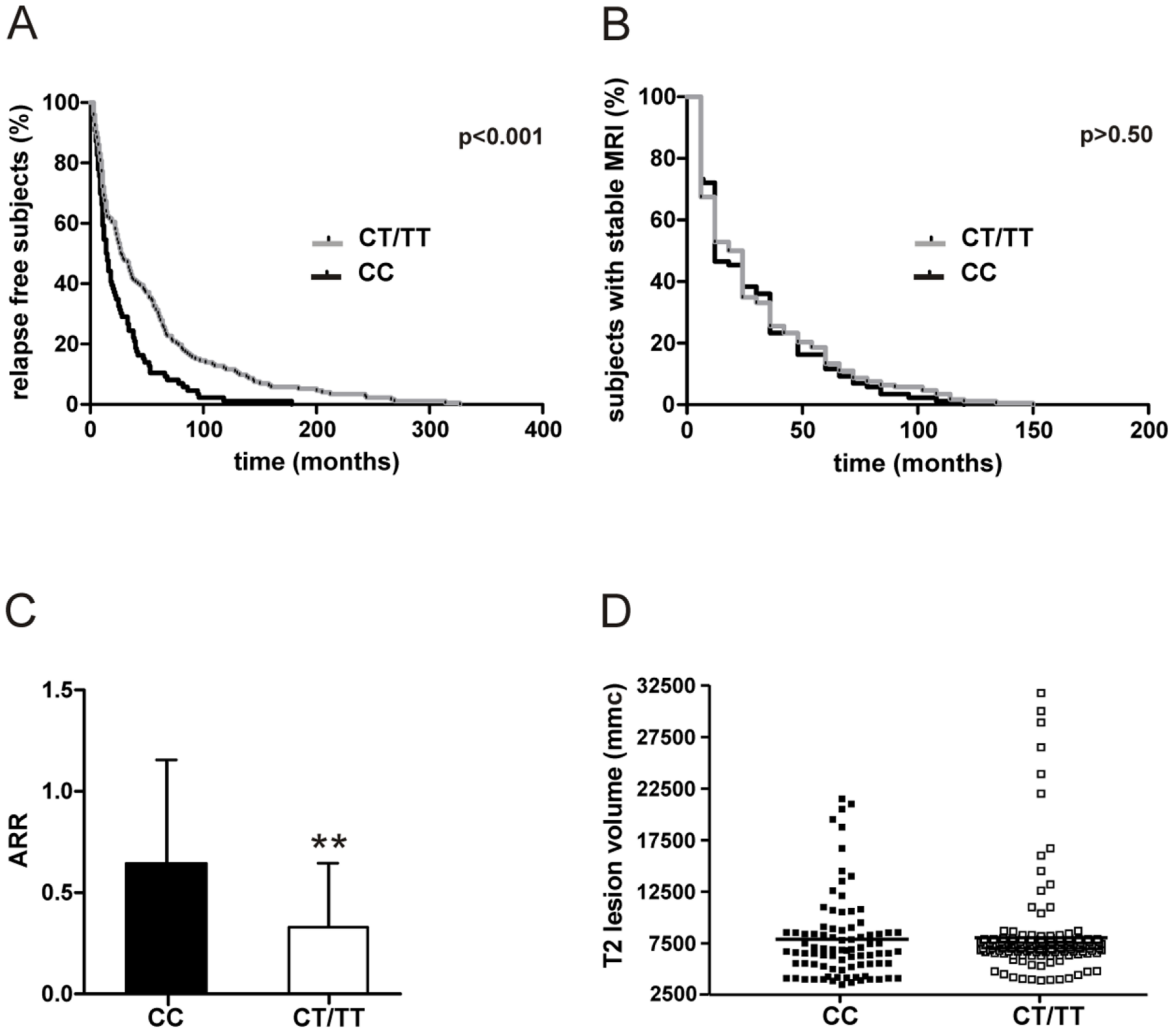
Effect of SNP rs4880213 on clinical and MRI measures of inflammatory activity in MS. A. The time to first relapse was lower among RR-MS with CC genotype. B. The time to detect inflammatory activity at MRI was similar between CC and CT/TT RR-MS patients. C. ARR, in the first three years of the disease, was lower among RR-MS patients with T allele. D. The graph shows that T2 lesion volume was similar between CC and CT/TT RR-MS patients. ** means p<0.01.

The opposite effects of rs4880213 polymorphism of GRIN1 gene in RR-MS and PP-MS could be explained by a role of NMDARs in the modulation of inflammation [22]–[24], since brain inflammatory activity is more severe in RR-MS than in PP-MS [12], [13]. We analyzed clinical and MRI indexes of inflammatory activity in RR-MS patients with at least five years of follow-up (n = 258, CC n = 86, CT/TT n = 172, no differences in term of sex, ages, disease duration) in order to evaluate this hypothesis. The mean time to first relapse was significantly lower in the CC group than in CT/TT group (26.2±29.4 months versus 52.3±62.1; p<0.001). Furthermore, the number of subjects with 2 or more clinical relapses in the first two years of the disease was higher compared to CT/TT group (35.0% versus 15.1%, p<0.001), although the number of subjects with MRI active scans was similar (40.7% versus 34.9%, p = 0.41). In line with this, a significant difference between the two groups was revealed by survival analysis considering the time to first clinical relapse (p<0.001; Fig. 3A) but not the time to detecting an active MRI scan (p>0.50; Fig. 3B). These data support the idea that rs4880213 T allele reduces the clinical impact of disease activity in RR-MS without affecting brain inflammation.

Furthermore, the annualized relapse rate (ARR) of the CT/TT group was significantly lower in the first three years of the disease but not later (3 years: CC 0.65±0.5, CT/TT 0.33±0.3, p<0.01; 5 years: CC 0.63±0.3, CT/TT 0.55±0.4, p = 0.11) while the MRI lesion load was not different at any time point (T2 lesion volume, 3 years: CC 7526.74±3712.5 mmc, CT/TT 8282.55±4497.8 mmc, p = 0.18; 5 years: CC 9436.04±4292.4 mmc, CT/TT 9588.32±5072.8 mmc, p = 0.81) (Fig. 3C,D), suggesting that an increase NMDAR-dependent neuronal plasticity is able to prevent the clinical manifestation of cerebral disease inflammation only at the early stage of the disease. However the percentage of relapses associated to 6 month-confirmed EDSS increase was lower in CT/TT group also at later stages (3 years: 8.2% versus 29.6%, p<0.001, n = 340; 5 years: 11.9% versus 32.1%, p<0.001, n = 748), confirming the role of rs4880213 T allele on disability recovery in RR-MS.

### rs4880213 T allele Preserves Neuronal Function in RR-MS and Induces Neuronal Damage in PP-MS

**Figure 4:**
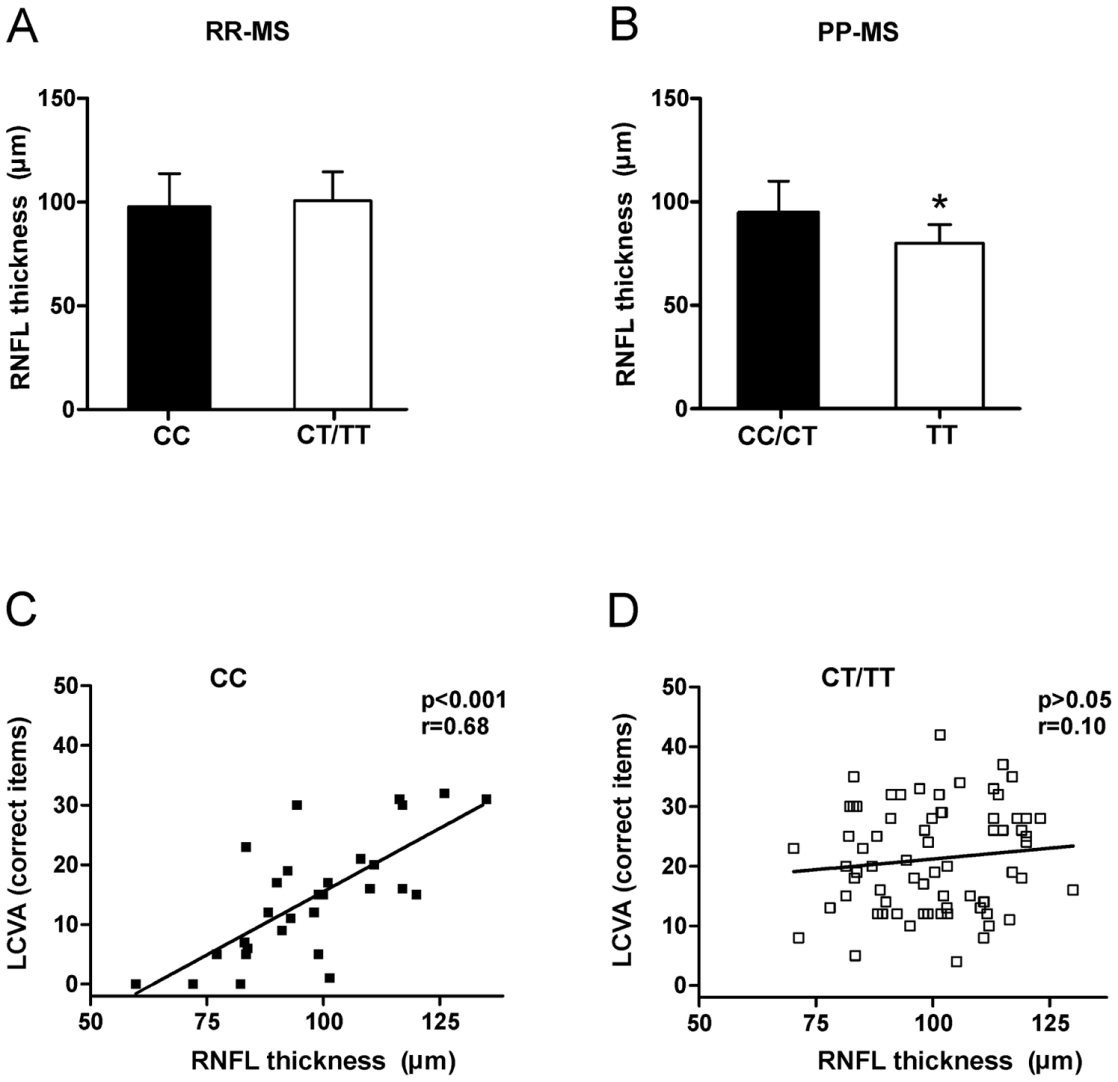
Effect of SNP rs4880213 on functional and morphological indexes of neuronal damage in MS. A. RNFL thickness was similar between CC and CT/TT RR-MS patients. B. RNFL thickness was lower among PP-MS subjects homozygous for T allele. C, D. Correlation analyses between RNFL thickness and LCVA in CC (C) and CT/TT (D) RR-MS patients. * means p<0.05.

The axonal and neuronal cell loss in MS has been convincingly associated with reduced RNFL thickness and macular volume (MV) at the OCT [25]–[28]. Thus, to confirm the idea that the increased glutamatergic transmission, associated to GRIN1 rs4880213 polymorphism, influences neurodegeneration in PP-MS while favors compensatory synaptic plasticity in RR-MS, we investigated the possible relationship between rs4880213 genotypes and OCT parameters in MS patients. Our data showed that neither MV nor RNFL thickness were altered in CC (n = 30) compared with CT/TT (n = 70) group among RR-MS subjects (MV: CC 7.04±0.6 mmc, CT/TT 7.09±0.5 mmc, p = 0.66; RNFL: p = 0.44, Fig. 4A), while they were significantly reduced in TT group (n = 22) among PP-MS patients, indicating increased neuronal damage (MV: CC/CT 7.03±0.5 mmc; TT 5.99±0.9 mmc, p<0.01; RNFL: p = 0.01, Fig. 4B). On the other hand, RNFL thickness values of RR-MS CC group were found to be positively correlated with Low-contrast visual acuity (LCVA) (p<0.001, r = 0.68; Fig. 4C), an emerging visual functional outcome [29]. Conversely, this correlation was lost in CT/TT group of RR-MS subjects (p = 0.40, r = 0.10; Fig. 4D), since also those with low RNFL thickness values scored well at LCVA test. In line with this, CT/TT group had better LCVA score than CC group (20.94±8.7 versus 14.53±9.8, p = 0.005). These findings are consistent with the idea that rs4880213 T allele favors the functional compensation to the subclinical axonal loss in the CNS of RR-MS patients.

### rs4880213 T allele Preserves Cognitive Function in MS

An alteration of NMDAR-dependent synaptic plasticity has been already related to cognitive impairment in MS and Alzheimer disease [30], [31]. To see if an increased recruitment of cognitive-related networks might represent a functional reserve with the potential to limit the severity of cognitive impairment, we performed cognitive tests in RR-MS and PP-MS patients with different rs4880213 genotypes. MS patients were, then, classified as cognitively impaired ([CI], n = 75) or cognitively preserved ([CP], n = 202) on the basis of their neuropsychological performance, without differences in terms of demographic characteristics (not shown).

The frequency of CP subjects and the cognitive impairment index (CII) mean values were not significantly different between CC (n = 18) and CT/TT group (n = 33) of RR-MS patients with maximum 3 years of disease (CP: CC 83.4%, CT/TT 81.8%, p = 1.0; CII: CC 1.67±1.8, CT/TT 1.54±2.2, p = 0.42). Interestingly, the mean values of PASAT and symbol digit modalities test (SDMT) were higher among CT/TT patients (PASAT: 52.18±15.4 versus 42.16±11.9, p<0.001; SDMT: 55.81±8.8 versus 44.5±7.8, p<0.001), indicating enhanced sustained attention and concentration.

**Figure 5:**
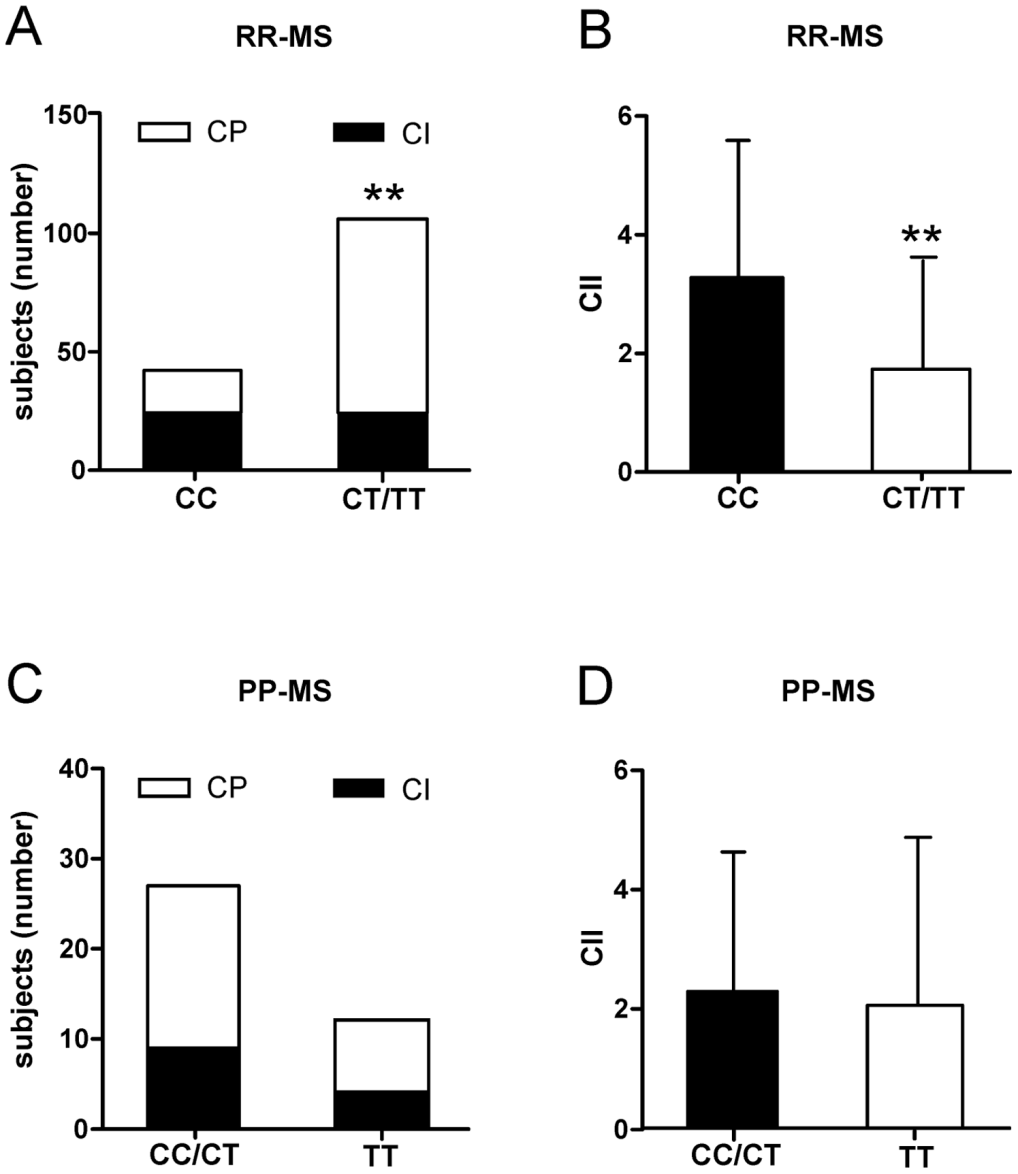
Effect of SNP rs4880213 on cognitive performances in MS. A. The graph shows that the frequency of cognitive preserved (CP) subjects, with at least five years of disease, was higher among RR-MS patients with T allele. B. The CII was lower among RR-MS patients with T allele. C. The graphs show that the frequency of cognitive impaired (CI) subjects was not higher among PP-MS subjects homozygous for T allele. D. The CII was not significantly higher among PP-MS subjects homozygous for T allele. ** means p<0.01.

Considering RR-MS patients with at least 5 years of disease duration (CC n = 42, CT/TT n = 106), the frequency of CP subjects was higher (77.4% versus 42.8%, p<0.001) and the value of CII was lower (p<0.001) in CT/TT group (Fig. 5A,B). They scored better at PASAT (49.04±10.5 versus 35.23±12.9; p<0.01), SDMT (53.68±6.7 versus 41.81±10.7 p<0.01), SRT-LTS (53.04±7.5 versus 44.04±12.3, p = 0.01) and SRT-CLTR (46.94±6.8 versus 37.95±13.3, p<0.01), indicating also preservation of verbal memory.

CII is negatively correlated with grey matter volume [32], [33]. However, in TT group of PP-MS patient, the CII and the frequency of CI subjects were not significantly higher respect to CT/CC group (p>0.1 for both comparisons; Fig. 5C,D), a front of a major extent of neuronal damage detected at OCT. PP-MS TT subjects had higher values at PASAT (51.37±9.5 versus 42.65±12.1, p<0.01) and SDMT (54.41±7.9 versus 47.85±8.1, p<0.01), whereas lower values were recorded at WLG (Word List Generation) (24.33±7.0 versus 29.68±3.5, p<0.01) and SRT-CLTR (35.00±15.7 versus 47.03±7.4, p<0.01), two tests strongly related to reduction of neocortical volume [32] and hippocampal atrophy [34] in MS.

## Discussion

Candidate gene studies with hypotheses driven by prior research remain a valuable approach for the definition of the role played by specific molecules and pathways in a given disease. Glutamate is essential for physiological synaptic transmission and plasticity, and is involved in a variety of pathophysiological mechanisms shared by acute and chronic disorders of the central nervous system, including MS [11], [22], [35]–[41]. Long-term potentiation (LTP) of the efficacy of glutamate synapses in unaffected areas is believed to mediate clinical recovery from focal brain lesions [1], [3]–[5], but excessive glutamate transmission is detrimental for neuronal survival, through a process termed excitotoxicity [6], [9]. Considerable overlap exists in the receptor and post-receptor mechanisms required for both LTP and excitotoxicity, and a plasticity-pathology continuum has in fact been proposed based on a plethora of experimental findings [42], [43]. In experimental stroke, for example, LTP mechanisms are favored by widespread up-regulation of NMDARs in regions adjacent to the ischemic brain lesion [44], and mediate both motor recovery [1], [45] and delayed neurodegeneration in the penumbra [43], [46].

Recent association studies have explored the role of NMDARs in the pathophysiology of a number of neuropsychiatric conditions, and have shown that specific SNPs of NMDAR subunits impact on the risk or the severity of these disorders. GRIN1 and GRIN2B polymorphisms, in particular, have been found to be associated with infantile spasms [47], neurodevelopmental disorders [48], Alzheimer’s Disease [49], Parkinson’s Disease [50], schizophrenia [51], obsessive-compulsive disorder [52], attention/deficit hyperactivity disorder [53], and bipolar disorder [54], confirming a widespread and essential role of NMDARs in brain function and dysfunction. In this study we explored the role of NMDARs in both plasticity and neurodegeneration mechanisms in MS, investigating the impact of four specific genetic variants of NMDAR subunits on a large cohort of RR- and PP-MS patients. Signaling through NMDARs is a key step in the induction of both synaptic plasticity and excitotoxic neuronal damage since both phenomena are prevented by pharmacological blockade of these receptors [55]–[58], and are favored by their enhancement [3], [59]–[62].

Our results show that the T allele of the rs4880213 SNP of NR1 subunit of NMDARs increases neuronal excitability measured by means of paired TMS, and is associated with better compensation of brain damage in RR-MS but with higher severity of PP-MS. These results are therefore consistent with the idea that potentiation of NMDAR-dependent excitatory synaptic transmission has a role in both adaptive plasticity and excitoxicity in MS patients. In agreement with this conclusion, results of a recent placebo-controlled clinical trial showed that MS patients treated with the NMDAR antagonist memantine experienced reversible neurological impairment [63]. On the other hand, it has been convincingly established that excessive synaptic signaling through NMDARs causes excitotoxic neuronal damage by altering mitochondrial activity [64]–[66] and in fact mitochondrial function is severely compromised in progressive MS [67]–[69].

The hypothesis that the T allele of the rs4880213 SNP of NMDAR increases glutamate transmission in MS patients relies on the evidence that cortical inhibition physiologically seen in paired TMS experiments is reduced in subjects homozygous for the T allele of this SNP, as expected for enhanced NMDAR function. Accordingly, contamination by glutamate-mediated facilitation limits the degree of cortical inhibition explored with paired TMS [70], and in fact drugs that block glutamate receptors increase this neurophysiological parameter [71]–[73]. Previous studies also support the conclusion of our TMS investigation, since the T allele of SNP rs4880213 has been associated with increased risk of Parkinson’s Disease [50], a neurodegenerative disorder with strong excitotoxic component [74]–[76], and with milder symptoms in schizophrenia [77], which is known to be associated with reduced NMDAR function [78], [79]. Of note, the region flanked by rs4880213 SNP of the GRIN1 gene is located near the 5′-upstream promoter sequence of the gene and is responsible for transcriptional regulation [80]. Although the exact molecular mechanism by which this SNP alters NMDAR function remains unknown, these data seem to imply different degrees of NMDAR expression in neurons, caused by different efficiency of gene transcription, in individuals carrying the T or C allele.

Enhanced NMDAR function was found to be particularly important for the preservation of cognitive abilities in our cohort of patients, a result that is in line with the role played by NMDARs and NMDAR-dependent synaptic plasticity in learning and memory processes [55], [56], [81]–[83]. Notably, not only RR-MS but also PP-MS carrying the T allele of rs4880213 SNP performed better at the PASAT and the SDMT, indicating residual NMDAR-dependent plasticity also in PP-MS. Of note, PASAT and SDMT explore cognitive domains frequently altered in MS, and poor performance at both tests has been associated with impaired NMDAR-dependent synaptic plasticity in MS patients [31].

The role of NMDARs in MS is however far more complex than postulated here, since expression of these receptors have been found not only on neurons but also on lymphocytes [84] and astroglia [85]. We have recently found a sensitization of NMDARs in experimental autoimmune encephalomyelitis (EAE), associated with dramatic activation of the astroglia, mytochondrial dysfunction and severity of the disease [86]. Previous studies demonstrated a role of NMDARs expressed on oligodendrocytes in demyelination and axonal loss in pathological conditions also including EAE and MS [10], [11], [39], [87]–[89]. Thus, it is likely that the different efficiency of NMDAR transmission in individuals carrying the T or C allele of rs4880213 SNP influence other critical aspects of MS pathophysiology, such as immune and glial response, contributing to explain at least in part our results.

Retinal alterations in MS patients accurately model the mechanisms of neurodegeneration in MS, and MV and RNFL thickness, obtained by OCT scans, are reliable measures of the integrity of, respectively, neurons and their axonal projections within the retina [26]. In fact, a close relationship has been found between RNFL thickness and brain atrophy and tissue damage evaluated at the MRI in MS subjects [90], [91]. Furthermore, patients with more progressive MS courses have more substantial RNFL loss [92], again confirming that the alterations of RNFL thickness mirror those occurring in the brains of individuals with MS. Based on these considerations, therefore, our evidence that both MV and RNFL thickness were significantly reduced in TT group among PP-MS patients indicates a role of NMDAR-mediated excitotoxic damage not only in the brain but also in the retina of PP-MS patients. In line with this conclusion, glutamate-mediated excitotoxicity has been implicated in the mechanism of neurodegeneration in experimental optic neuritis [93], again supporting the common pathophysiology of neuronal damage in the brain and in the retina of MS patients.

The difference between the discovery and replication cohorts in terms of demographic and clinical features could be a limitation of this study. However, the significance of our results was analyzed performing multiple logistic regressions, in which counfonding factors were taken into account. Furthermore, the replication of the association between the C allele of rs4880213 SNP and disability progression for different levels of EDSS strengthens the validity of our data.

In summary, the results of the present study indicate a complex role of NMDARs in MS, and provide potentially useful information for the optimization of symptom-relieving and neuroprotective strategies in this disabling neurological disorder.

## Methods

This study complied with the principles of the Declaration of Helsinki, and was approved by the local Ethical Committees. All the subjects gave their written informed consent to the study.

### Subjects

A total of 2239 subjects of European ancestry were included in this study. All the cases had a diagnosis of MS according to revised McDonald criteria [94], including 177 with a primary progressive course. Of them, 691 were central-southern Italian. Blood sample collection and the clinical assessments were performed at the MS Centers of the Tor Vergata University Hospital of Rome and of University of Cagliari by MS specialist neurologists. The remaining subjects were from United States and enrolled at the Department of Neurology of California University in San Francisco, to replicate the primary analysis.

Demographic and clinical information were derived from medical records. MS disease onset was defined as the first episode of focal neurological dysfunction indicative of MS. Disease duration was estimated as the number of years from onset to the last assessment of disability. Only patients with at least one year of follow-up were enrolled.

The BREMS score was calculated, using gender, age of onset and clinical events of the first year of disease, to identify individual risk of secondary progression [21] for RR-MS patients when all the information was available (n = 384).

At the time of diagnosis, all RR-MS patients had started disease-modifying therapy (glatiramer acetate 20 mg s.c. every day or interferon beta 1a 44 mcg s.c. three times weekly or interferon beta 1b 250 mcs s.c. every other day). Relapses were defined as the development of new or recurrent neurological symptoms not associated with fever or infection lasting at least 24 h. The ARR was defined as the number of relapses per year. In addition, the number of relapses in the first two years of the disease, the time to the first relapse and the occurrence of relapses associated to sustained disability, were used to define disease severity.

### Disability Assessment

Disability was determined by a specially trained (Neurostatus training and documentation DVD for a standardized neurological examination and assessment of Kurtzke’s functional systems and Expanded Disability Status Scale for MS patients. Basel, Switzerland: Neurostatus, 2006. Available at http://www.neurostatus.net) and certified examining neurologist using EDSS, a 10-point disease severity score derived from nine ratings for individual neurological domains [95]. The EDSS, evaluated every six months since the disease onset, was used in combination with disease duration to calculate two measures of disease severity, the PI and the MSSS. PI was defined as EDSS/disease duration. The MSSS is an algorithm that relates EDSS scores to distribution of disability in patients with comparable disease durations [96]. MSFC [97] scores were obtained on the same day as the EDSS scores in a subgroup of RR-MS (n = 384) and PP-MS patients (n = 78). This composite test consists of three domains, scoring ambulation (TWT), upper extremity function (9HPT), and cognition (PASAT) in separate measurements. These separate quantitative scores were used in our analyses. Disability progression using the MSFC was defined as a sustained change of ≥20% from baseline for any of the 3 components of the MSFC. Worsening was required to persist for >6 months.

EDSS and MSFC scores were taken in account for the assessment of disability progression when obtained at least 30 days since stabilization/resolution of previous relapse and/or corticosteroid treatment.

### Neuropsychological Assessment

Cognitive function was assessed through the Brief Repeatable Neuropsychological Battery (BRB) [98] by an expert trained clinician in a subgroup of remitting MS patients (n = 199) and PP-MS patients (n = 78). Patients were tested after at least 3 months from previous relapse and/or detection of active scans at MRI. The BRB assesses the cognitive domains most frequently impaired in MS [99], [100] and incorporates tests of verbal memory (Selective Reminding Test [SRT]); visual memory (10/36 Spatial Recall Test); attention, concentration, and speed of information processing (PASAT, SDMT); and verbal fluency (WLG). Moreover, the stroop word-colour task (ST) was administered to evaluate frontal lobe executive functions, which are not assessed by the BRB. A 100-item version of the Stroop Test was applied [101].

Performance on each test of the BRB and ST was assessed by applying the available Italian normative values [100]. Failure of a test was defined when the score was at least 2 standard deviations below the mean normative values.

Those patients who failed at least two tests were considered CI and those who failed less than two tests were considered CP. A grading system was applied to each patient’s score on each cognitive test, dependent on the number of standard deviations (SDs) below the normative mean (0: patient scored at or above normative mean; 1: patient scored ≤1 SD below normative mean; 2: patient scored >1 SD, but ≤2 SD below normative mean, etc.). The sum of these grades was determined across all variables to give the CII, a single overall measure of cognitive impairment for each patient [102]–[104].

No subject was taking psychoactive drugs or substances that might interfere with neuropsychological performance.

### Ophthalmologic Assessment

Medical history with respect to visual symptoms was taken from all MS subjects. Self-report and physician report were confirmed by record review.

A subset of RR-MS patients (n = 100) and PP-MS patients [75] without history of optic neuritis and ophthalmological disease underwent measurement of RNFL thickness and MV for both eyes using Stratus OCT™ Optical Coherence Tomography (software version 4.0.2, Carl Zeiss Meditec, Inc.) [105]. Briefly, for MV, retinal thickness was measured automatically as the distance between the vitreoretinal interface and the anterior boundary of the retinal pigment epithelium. Stratus OCT images were generated using the fast map scan protocol consisting of six radial scans spaced 30° apart, with each scan measuring 6 mm in length. Each image had a resolution of 10 µm axially and 20 µm transversally. All Stratus OCT images had a signal strength of 6 µm. RNFL thickness measurements were read from the automated measurements generated by the machine using the Fast RNFL analysis. For the study scanning was performed after pharmacological dilation. Average RNFL thickness for 360° around the optic disc was recorded. Values were adjusted for age. One randomly chosen eye from each subject was included in the study. LCVA testing was performed for each eye separately using retroilluminated low-contrast Sloan lettercharts (1.25% contrast at 2 m). Testing was performed by trained technicians experienced in examination of patients for research studies, and patients wore their habitual glasses or contact lenses for distance correction.

### Single and Paired TMS

A group of MS patients underwent single and paired TMS to measure the impact of NMDAR SNP variants on cortical excitability [17]. Electromyographic (EMG) traces were recorded from the first dorsal interosseous (FDI) muscles of the left hand with 9-mm diameter, silver–silver chloride (Ag–AgCl) surface cup electrodes. The active electrode was placed over the muscle belly, and the reference electrode was placed over the metacarpophalangeal joint of the index finger. Responses were amplified with a Digitimer D360 amplifier (Digitimer, Welwyn Garden City, Hertfordshire, United Kingdom) through filters set at 20 Hz and 2 kHz with a sampling rate of 5 kHz, then recorded by a computer with SIGNAL software (Cambridge Electronic Devices, Cambridge, United Kingdom).

MEPs were evoked through a figure-of-eight coil with external loop diameter of 70 mm connected to a Magstim 2002 magnetic stimulator (Magstim Company, Whitland, Wales, UK). The hand motor area of right M1 was defined as the point where stimulation evoked the largest MEP from the contralateral FDI muscle. The motor hot spot was identified at the beginning of each experimental session and marked over the patients scalp with a pencil. The coil was held tangentially to the scalp surface with the handle pointing posteriorly and laterally at about 45° with respect to the mid-sagittal axis of the head. We defined the RMT as the lowest intensity that evoked five small responses (approximately 50 µV) in the contralateral FDI muscle in a series of 10 stimuli when the subject kept the FDI muscles relaxed in both hands, according to international standards. AMT was defined as the lowest intensity that evoked five small responses (about 200 µV) in a series of 10 stimulations when the subject made a 10% of maximal voluntary contraction. Measurements were made on each individual trial and the mean peak-to-peak amplitude of the conditioned MEP was expressed as a percentage of the mean peak-to-peak amplitude of the unconditioned test pulse.

Paired-pulse TMS was used to assess SICI and ICF mediated by both intrinsic GABAAergic or excitatory circuits [106] of the right M1.

To assess SICI/ICF, a sub-threshold conditioning stimulus (CS), delivered 2 and 3 ms (for SICI) or 10 and 15 ms (for ICF) prior to the test stimulus (TS), was used to excite M1 intracortical inhibitory or excitatory fibers, and the subsequent reduction or potentiation in contralateral MEP amplitude compared to the non-conditioned MEP provided a measure of SICI or ICF, respectively. The CS was delivered at 80% of right AMT [70], [106]. For all paired TMS experiments ten non-conditioned MEPs and ten conditioned MEPs, at each ISI, were collected in a randomized order at a rate of 0.2 Hz. The intensity of the TS corresponded to the intensity required to elicit MEPs of 1 mV peak-to-peak mean amplitude in the relaxed FDI. MEP latency was determined as the time elapsed from the TMS stimulus artifact to the onset of the mean non-conditioned MEPs, expressed in ms.

### MRI

MRI scans (1.5 Tesla), consisted of dual-echo proton density, FLAIR, T2-weighted spin-echo images (T2-WI) and pre-contrast and post-contrast T1-weighted spin-echo images (T1-WI), were analyzed by a neuroradiologist who was unaware of the patient's clinical details (31). A new Gd+ (0.2 ml/Kg e.v.) lesion was defined as a typical area of hyperintense signal on postcontrast T1-WI. A new or newly enlarging lesion on T2-WI was defined as a rounded or oval lesion arising from an area previously considered as normal appearing brain tissue and/or showing an identifiable increase in size from a previously stable-appearing lesion. An active scan was defined as showing any new, enlarging or recurrent lesion(s) on postcontrast T1- and T2-WI. T2 lesion volume was determined by manual tracing.

### Determination of Single Nucleotide Polymorphisms in the GRIN1 and GRIN2B Genes

The MassARRAY Assay Design 3.1 software was used to design a single 20-multiplex reaction in which the two SNPs rs4880213 and rs6293 of GRIN1 gene and the two SNPs rs7301328 and rs1805247 of the GRIN2B gene were included. Genotyping was performed using iPLEX Gold technology [17], [107] and MassARRAY high-throughput DNA analysis with Matrix-assisted laser desorption/ionization time-of-flight (MALDI-TOF) mass spectrometry (Sequenom, Inc., San Diego, CA), according to manufacturer’s instructions. The four SNPs of the NMDA receptor showed a call rate higher than 95%, with no significant departure from Hardy-Weinberg equilibrium.

### Statistical Analysis

We assessed the impact of the four polymorphisms located in genes coding for NMDAR subunits on neurological disability of our group of MS subjects with two different models. First, we evaluated in the discovery dataset the association of each single SNP with the EDSS by means of the Kruskal-Wallis rank sum test. The test is the extension of the Wilcoxon rank sum test when the grouping factor has more than 2 levels (e.g., the three possible genotypes CC, CT, TT). We used a non parametric test because the distribution of the EDSS was not Gaussian. We replicated the analysis for each SNP, and corrected the p-values with the Holm method [108].

Then, we applied a multiple logistic regression in order to assess the effect of clinical and genetic predictors on the disability of Italian MS patients (discovery dataset). We estimated the degree of disability by means of the dichotomous EDSS (cut-off point of 2.0, at which clinical disability starts to be appreciated). Four clinical variables (years of disease, age at the blood sample, gender and onset type), the site of recruitment, and 4 SNPs were included as predictor variables. The gender (M for male or F for female), the onset type (R for relapsing or P for progressive), and the site of recruitment (Cagliari or Rome) were all coded as dummy variables, as specified in the Table 2.

For each SNP, we used the number of copies of the minor allele as the predictor variable. The minor allele is T in rs4880213; C in rs7301328; G in rs1805247; G in rs6293. This coding schema assumes that the predicted outcome is a monotonic function of the number of copies of the allele, and it has been previously applied in similar studies (e.g., 109). The assessment of the association between significant SNPs and EDSS was replicated in an independent sample (replication dataset). The significance of each parameter was tested with both the Wald statistics and the Likelihood Ratio test [110]. Finally, Holm correction was applied to correct for multiple testing.

The model was fitted via penalized maximum likelihood (according to [18]) in order to override a problem of quasi-complete separation for the covariate onset type. Sombekke et al. [111] applied a similar analysis. In their article, they compared the logistic, linear and Cox regression and reported that, with respect to their dataset, logistic regression had the highest predictive power. Sombekke et al. [111] used the MSSS as response variable. It is worth noting that, differently from the MSSS, the EDSS is expected to correlate with the duration of the disease. For this reason the duration of the disease (in years) was also included as predictor variable in our multiple regression analysis.

We chose the EDSS value of 2.0 as cut-off point of early clinical disability because of sample distribution, only a minor part of the discovery data set having higher EDSS scores. In order to confirm the result for an higher value of EDSS, we pooled the discovery and the replication data sets and replicated the analysis using cut-off point of EDSS 4.0, at which restriction in ambulation starts to be appreciated.

Finally, the association between significant SNPs and EDSS was assessed in the subgroup of subjects with progressive onset, using EDSS>4.0 as cut-off point value.

In addition, mean values were calculated for each clinical and severity variables, stratifying by genotype. In these analyses, comparisons between two groups of data were performed by Student’s T Test, while multiple group comparisons were performed by ANOVA followed by Tukey HSD.

The association between the proportion of subjects and the presence of other variables (BREMS score ≥2, BREMS score ≤-0.63, active MRI, >2 relapses, sex) were analyzed by using Fisher exact test. Correlation analysis was performed by calculating Spearman coefficients.

Survival curves on the time to first clinical relapse and the time to an active MRI scan were analyzed using Log-rank (Mantel-Cox) Test. Patients who did not reach the endpoint were considered as censored data.

Analysis of TMS data was performed using a repeated measures ANOVA on the normalized data with genotype as between-subjects and ISI as within-subject main factors.

Data were presented as mean ± SD. The significance level was set at p<0.05.

We used R software 2.15.3 (R Development Core Team, 2013) for the univariate and multiple regression analyses. Specifically, we used the function brglm (from the R package brglm) in order to fit the logistic regression. Prism 5.0 (GraphPad Software, Inc., CA USA) was used for the other analyses.
